# Acoustic Cardiography Captures Stress-Dependent Ventricular Dysfunction and Distinguishes Heart Failure Phenotypes in a Porcine Model

**DOI:** 10.3390/biomedicines14081669

**Published:** 2026-07-24

**Authors:** Francesco Paolo Lo Muzio, Lorenzo Fassina, Jens Ötvös, Katharina Wierling, Sebastian Baer, Leonhard Berboth, Andreas Kind, Alessandro Faragli, Alessio Alogna

**Affiliations:** 1 Department of Medicine and Surgery, University of Parma, Via Gramsci 14, 43126 Parma, Italy; francescopaolo.lomuzio@unipr.it; 2 Department of Cardiology, Angiology and Intensive Care Medicine, Deutsches Herzzentrum der Charité, Campus Virchow-Klinikum, Augustenburgerplatz 1, 13353 Berlin, Germany; jens.oetvoes@dhzc-charite.de (J.Ö.); katharina.wierling@charite.de (K.W.); sebastian.baer@charite.de (S.B.); andreas.kind@charite.de (A.K.); alessandro.faragli@dhzc-charite.de (A.F.); 3 Department of Electrical, Computer and Biomedical Engineering, University of Pavia, Via Ferrata 5, 27100 Pavia, Italy; 4 DZHK (German Centre for Cardiovascular Research), Partner Site, 10785 Berlin, Germany

**Keywords:** HFpEF, HFrEF, porcine model, invasive hemodynamics, electronic stethoscope, machine learning

## Abstract

**Background**: Heart failure with preserved ejection fraction (HFpEF) is a heterogeneous syndrome requiring invasive and/or exercise stress testing for diagnosis, in contrast with the homogeneous nature of HF with reduced EF (HFrEF). We previously demonstrated that acoustic cardiography (AC), combined with advanced signal analysis, can non-invasively estimate left ventricular (LV) functional indices in an experimental swine model of HFrEF. **Objectives**: To evaluate whether AC-derived features capture stress-dependent LV functional changes, supporting phenotypic differentiation between porcine models of HFpEF, HFrEF, and Control conditions. **Methods**: Synchronized invasive LV-pressure and non-invasive ECG, pulse oximetry, and AC signals were collected from 12 anesthetized, closed-chest Göttingen minipigs (Control, *n* = 3; HFrEF, *n* = 5; HFpEF, *n* = 4). Through signal analysis, we derived time and frequency features from the non-invasive signals to predict, using our AI model, the invasively measured LV functional indices. Atrial pacing was performed up to 160 bpm as a controlled heart-rate stress paradigm. Two AI modeling strategies were employed: a standard 80/20 train/test ratio, and a leave-one-animal-out (i.e., 1 animal per health status) to assess generalizability. **Results**: Standard blind testing achieved >95% accuracy in phenotype classification with <3% relative error for predicted LV indices. The leave-one-animal-out classification performance remained robust (79–95% accuracy), supporting translational potential despite inter-animal variability. Notably, HFpEF animals exhibited greater variability in AC-features across increasing heart rates compared to Control and HFrEF. **Conclusions**: AC-based modelling offers a rapid, non-invasive approach for assessing LV function. Our methodology may complement existing diagnostic tools, particularly for conditions like HFpEF, but warrants further validation in clinical populations.

## 1. Introduction

Heart failure (HF) is a heterogeneous clinical syndrome characterized by symptoms (e.g., dyspnea, exercise intolerance) and/or signs (e.g., edema, jugular venous distension) arising from structural and/or functional cardiac abnormality, supported by elevated natriuretic peptide levels and/or objective evidence of pulmonary or systemic congestion [1]. This definition acknowledges substantial diversity in underlying pathophysiological mechanisms across HF phenotypes, although ejection fraction (EF) remains the primary echocardiographic parameter used to distinguish HF with preserved EF (HFpEF, EF ≥ 50%) and HF with reduced EF (HFrEF, EF < 40%).

HFrEF is characterized by impaired systolic function and reduced cardiac output reserve, with relatively consistent structural and functional abnormalities, readily captured by standard imaging modalities [2]. In contrast, HFpEF is characterized by impaired ventricular relaxation, increased chamber stiffness, and abnormal ventricular–vascular coupling, frequently driven by systemic comorbidities such as obesity, dyslipidemia, and hypertension [3]. Diagnosis and phenotyping of HFpEF remain challenging because no single non-invasive parameter is sufficient. Instead, the diagnosis relies on the integration of clinical findings, natriuretic peptide concentrations, structural and functional echocardiographic abnormalities, and, when required, invasive evidence of elevated cardiac filling pressures [4]. In some cases, symptoms and hemodynamic abnormalities manifest under physiological stress, requiring exercise testing and/or invasive hemodynamic assessment [5], which obviously cannot be performed in all patients [6,7]. Accordingly, non-invasive approaches capable of capturing stress-dependent cardiac function are highly desirable.

In this context, acoustic cardiography (AC) has re-emerged as a promising non-invasive modality for interrogating cardiac mechanics, particularly when combined with modern signal processing approaches, such as artificial intelligence (AI) techniques for the study of heart sounds [8,9]. Previous studies have demonstrated that AC-derived features contain clinically relevant information in a range of cardiovascular conditions such as pulmonary arterial hypertension [10], atrial fibrillation [11], coronary artery disease [12], and HF [13,14]. We previously demonstrated that a multimodal approach integrating AC, electrocardiography (ECG), and pulse oximetry (POX) may aid a non-invasive estimation of left-ventricular-pressure-derived indices in an experimental porcine HFrEF model [15]. Importantly, our mathematical model does not focus on the recognition of specific heart sounds, but it leverages a multimodal approach integrating both temporal and frequency features from ECG, POX, and AC to capture broader mechanical signatures of cardiac function [15].

In this study, we investigated the applicability of our previous AC-derived modeling approach for capturing the stress-dependent LV function in a clinically relevant porcine model of cardiometabolic HFpEF [16]. Furthermore, we assessed whether these features support the phenotypic differentiation between HFpEF, HFrEF, and Control conditions under increasing heart-rate stress. By integrating invasive hemodynamic measurements with non-invasive multimodal signals, this work explores the potential of AC as a tool for the non-invasive functional phenotyping of heart failure. This study is intended to support functional characterization rather than to replace established diagnostic pathways and protocols.

## 2. Materials and Methods

### 2.1. Porcine Models of HFrEF and HFpEF

Female Göttingen minipigs (Ellegaard Göttingen Minipigs, Dalmose, Denmark) underwent different established protocols to induce HFrEF and cardiometabolic HFpEF. In detail, the minipigs underwent continuous right ventricular tachypacing for a total of 6 weeks, with 2 weeks at 180 bpm and 4 weeks at 200 bpm as previously described [17,18]. Chronic compensated HF with a highly depressed EF (i.e., <40%) was confirmed by echocardiography already after 4 weeks of tachypacing. This experimental protocol (G0064/19) was approved on 19 August 2019 by the Landesamt für Gesundheit und Soziales Berlin (LAGeSo, Berlin, Germany) and conformed to the Guide for the Care and Use of Laboratory Animals published by the US National Institute of Health (NIH Publication No. 85-23, revised 1996). All the temporal signals (ECG, POX and AC) required for our prediction models were recorded in five (*n* = 5) HFrEF minipigs from this trial and were consequently included in this work.

Four (*n* = 4) female Göttingen minipigs underwent the protocol described by Sharp et al. [16]. Briefly, 14-month-old animals were subcutaneously implanted with drug-eluting chips to continuously release deoxycorticosterone acetate (DOCA, 50 mg/kg of body weight) for 60 days. From that day, the animals were also fed a diet high in cholesterol, fat, fructose, and salt over 20 weeks (S8090-S302, SM “Ossabaw”, SNIFF, Soest, Germany). This two-hit model reproducibly induces cardiometabolic HFpEF, with obesity, hypertension, and preserved EF (i.e., ≥50%). In our small cohort, the HFpEF phenotype was defined by the combination of preserved LV EF, increased LV filling pressure exceeding a 15 mmHg threshold (by direct measurement of LV EDP), and concentric LV remodeling as reflected by increased relative wall thickness (RWT). This experimental protocol (G0005/22) was approved 18 July 2022 by LAGeSo and conformed to the Guide for the Care and Use of Laboratory Animals.

Control animals (*n* = 3) consisted of Göttingen minipigs without tachypacing and without exposure to the cardiometabolic HFpEF protocol, i.e., fed a standard diet (V 4173-000, Minipig, energy reduced, SNIFF, Soest, Germany), serving as physiological reference subjects. Control animals from each trial were included to provide the baseline physiological ranges rather than to represent a population-based healthy cohort.

Animals from each experimental group were studied at the end of the respective trial under identical anesthesia conditions, conforming to dosing guidelines. This approach ensured that, while the absolute acoustic signals could be altered, the relative differences and features analyzed by our AI model remained consistent across subjects within the cohort.

### 2.2. Cardiac Catheterization and Signal Acquisition

LV catheterization was performed in Control, HFrEF and HFpEF minipigs at the end of their respective protocols before euthanasia as previously described [19]. Briefly, anesthesia was induced with administration of 1 mg/kg propofol and continued after intubation with 1–1.5% isoflurane, 20 µg/kg/h fentanyl, and 0.2 µg/kg/h pancuronium. The animals were ventilated (Cato Dräger Medical, Lübeck, Germany) with a fraction of inspired oxygen of 0.5, an I:E ratio of 1:1.5, a positive end-expiratory pressure of 5 mmHg, and a tidal volume of 10 mL/kg. Heart rate (HR), arterial blood pressure, and arterial oxygen saturation were continuously monitored and a body temperature of 37.5–38.5 °C was carefully preserved. A balanced crystalloid infusion (Elo-Mel Isoton, Fresenius Kabi, Graz, Austria) was administered at a fixed rate of 10 mL/kg/h until euthanasia. Animals were acutely instrumented closed chest under fluoroscopic guidance with a LV conductance catheter (5F, 12 electrodes, 7 mm spacing; MPVS Ultra, Millar Instruments, Houston, TX, USA) as previously described [18]. A quadripolar stimulation catheter was advanced into the right atrium (9F SafeSteath II, Oscor Inc., Palm Harbor, FL, USA; CapSureFix Novus, MRI SureScan 5076, Medtronic, Minneapolis, MN, USA) to perform the pacing via an external stimulator (UHS20, Biotronik, Berlin, Germany) with steps of 20 bpm up to a maximum of 160 bpm. These pacing steps provide a controlled heart-rate stress paradigm that captures key aspects of cardiac functional reserve without reproducing the full systemic effects of exercise.

Simultaneously with invasive LV pressure measurements [mmHg], non-invasive ECG, AC, and POX signals were acquired via LabChart 10 software (ADInstruments Ltd., Oxford, UK) at a sampling frequency of 1 kHz throughout the protocol as follows: (i) ECG signal [V], using a standard 3-lead ECG; (ii) AC [mmHg] obtained via a microphone (MLT201 Cardio Microphone, ADInstruments) attached to the thoracic skin at the level of the LV apex; (iii) POX signal [mmHg] acquired with a tail-cuff.

After completing the right atrium pacing protocol, the animals were euthanized by intracardiac administration of potassium chloride, in accordance with the institutional ethical guidelines.

### 2.3. Signal Processing and Feature Extraction

The processing of the acquired temporal signals was described in detail in our previous work [15]. Briefly, at least 3 min with stable hemodynamic conditions (i.e., with less than 10% variation in systemic pressure) were extracted from the recording (i.e., spontaneous heart rate or baseline, 100, 120, 140 and 160 bpm) of each signal. A custom calculus script written in MATLAB^®^ language (The MathWorks, Inc., Natick, MA, USA) detected the HR on the ECG and then up- and down-enveloped the MIC signal corresponding to that recorded pacing cut; the difference between the two envelopes was calculated and called Microphone Envelope Difference signal (MED, mmHg), which was later bandpass-filtered using the detected HR. Then, the MED signal was employed to approximate the temporal boundary between systole and diastole of consecutive beats independently of the LV pressure signal. Consequently, each heartbeat was segmented into diastole and systole via ECG analysis to obtain: (i) the Left Ventricle end-diastolic pressure (LV EDP, mmHg), the end-systolic pressure (ESP, mmHg), and the maximum rate of pressure rise (dP/dt_max_, mmHg/s), which are the LV function indices to be predicted; (ii) the multiplicative inverse of the pulse transit time (invPTT), calculated from the ECG and POX signals. Additionally, for each heartbeat and separately for diastole and systole, the melody spectrum (MEL) of the MIC signal was analyzed via the MATLAB^®^ Audio Toolbox™ to compute two sets of predictors: set A: spectral flux, kurtosis, skewness, and slope [20,21,22,23]; set B: 13 MEL frequency cepstral coefficients in 2nd derivative (delta-delta-MFCCs) [24,25,26,27,28].

To recapitulate, we combined time and frequency features (i.e., invPTT, set A, and set B) for a total of 18 predictors used in the linear regression models. Based on physiological timing relationships, as an overall strategy, the systolic sounds were employed to predict the precedent LV EDP and the contemporary dP/dt_max_, whereas the diastolic sounds were employed to predict the precedent ESP. Approximately 500 cardiac cycles per animal were included in the analysis. The animal represented the unit of biological replication. Linear regression models were constructed and evaluated using the *fitlm* function from the Statistics and Machine Learning Toolbox in MATLAB (v. R2024b, MathWorks, Inc.) which provides the “whole model” (i.e., the predicting features condensed in their linear combination), the R^2^ parameter, and the RMSE (root mean square error).

### 2.4. Statistical Analysis

Normal distribution of both echocardiographic and hemodynamic data was assessed with the Shapiro–Wilk test. Accordingly, the one-way ANOVA followed by the Šídák post hoc test or the Kruskal–Wallis test followed by Dunn’s tests were employed as appropriate. Data in the text is reported as mean ± standard deviation. GraphPad Prism (version 10.0.0 for Windows, GraphPad Software, Boston, MA, USA, www.graphpad.com) was used for this comparison between groups (Control, HFrEF and HFpEF). Because this work represents a secondary analysis of two distinct animal trials, the cohort size was strictly limited to those subjects in whom flawless, simultaneous acquisition of all four high-fidelity continuous signals (i.e., MIC, ECG, POX, and LVP) was successfully achieved. Therefore, an a priori power analysis was not performed before this work.

To assess potential multicollinearity among the independent variables, a preliminary screening was conducted using Pearson’s correlation. Overall, no strong pairwise correlations were observed, suggesting that immediate pairwise redundancy was not a major concern for the model (Supplemental Figure S1). However, a limited number of acoustic features show collinearity but they are usually used together to describe an acoustic phenomenon so we opted not to exclude them a priori. More importantly, the invPTT (arising from ECG and POX signals) was found not to be correlated with the acoustic features.

Due to the unbalanced group sizes and repeated measurements within animals, the estimated marginal means (EMMs) were calculated to enable a fair comparison across phenotypes and heart-rate conditions using the AC predictors of both diastole and systole plus the invPTT (i.e., a total of 35 predicting features). The EMMs provide a fairer comparison as they are the mean outcome for a specific group adjusted by holding constant all other variables in the model at the mean of the covariates. In detail, we have performed a multivariate repeated measures ANOVA with the estimated marginal means followed by the Tukey–Kramer post hoc test, thus isolating the effect of the primary factor of interest (i.e., the clinical, health status or the HR) from the confounding influence of other variables. An α of 0.05 was elected as significant. GraphPad Prism (version 10.0.0 for Windows, GraphPad Software, Boston, Massachusetts, USA, www.graphpad.com) was used for this comparison between groups. Statistical analyses were performed with the animal as the independent experimental unit.

### 2.5. Artificial Intelligence Classifiers

Multiple supervised machine learning classifiers were systematically evaluated (trained, validated and tested) via the Classification Learner app (v. R2024b MATLAB^®^, Statistics and Machine Learning Toolbox™), to assess whether acoustic cardiography-derived features contain sufficient information to distinguish the heart failure phenotypes (Visual Abstract). In detail, to classify an observation into one of the three groups, we employed all the features of the diastolic and systolic cardiac sounds plus the invPTT (i.e., a total of 35 predicting features). To prevent overfitting, we utilized 80% of the dataset for model training with a 10-fold cross-validation, whilst the remaining 20% of the data were reserved for blind testing. The results are expressed in terms of validation accuracy (%) and test accuracy (%). For reproducibility, all the hyperparameters of the AI models are also reported. Additionally, we trained and validated the AI models employing the leave-one-out strategy (i.e., 1 animal per health status was not used to train and validate the models), in order to simulate a translational scenario in which the phenotypic classification is performed for an unseen subject.

Overall, this methodological framework was designed to explore non-invasive functional phenotyping under controlled conditions rather than to establish a diagnostic algorithm.

## 3. Results

### 3.1. Heart Failure Models’ Characteristics

**Figure 1:**
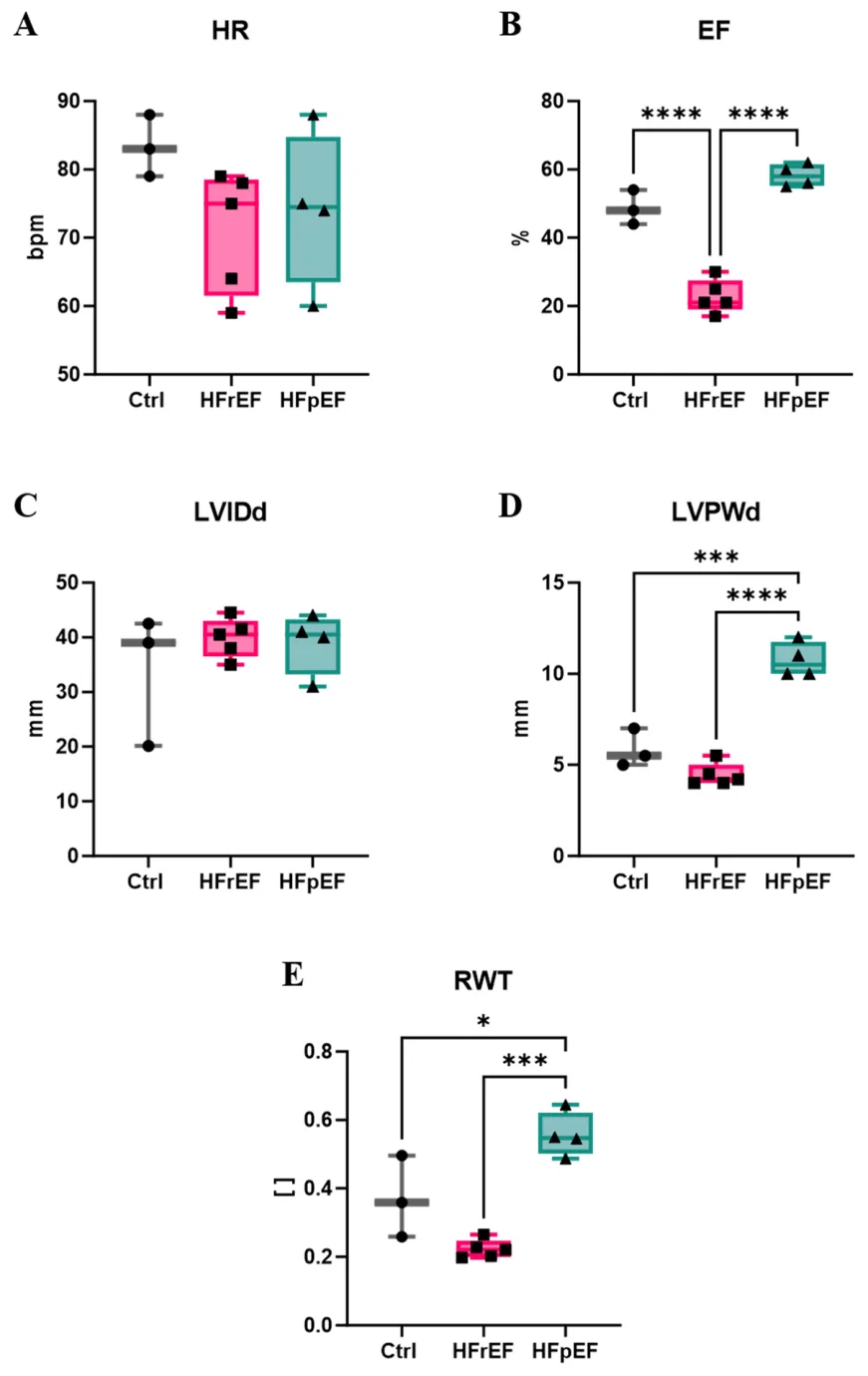
Echocardiographic comparisons of HFrEF and HFpEF porcine models. Boxplot of the echocardiographic parameters: (**A**) heart rate (HR); (**B**) ejection fraction (EF); (**C**) left ventricular internal diameter at diastole (LVIDd); (**D**) left ventricular posterior wall at diastole (LVPWd); and (**E**) relative wall thickness (RWT). Comparisons are shown between healthy controls (Ctrl, grey), HFrEF (pink), and HFpEF (teal) minipig models. No significant differences were observed in HR or LVIDd across the three groups. The HFrEF model is characterized by a significant reduction in ejection fraction compared to the HFpEF group. Conversely, the HFpEF model displays significant concentric hypertrophy, indicated by an increased LVPWd compared to the HFrEF group and thickened walls relative to chamber size (i.e., increased RWT). Data are expressed as median and interquartile range (25th–75th percentiles) with individual data points plotted. One-way ANOVA followed by the Šídák post hoc test for multiple comparisons. * *p* < 0.05, *** *p* < 0.001, **** *p* < 0.0001.

**Figure 2:**
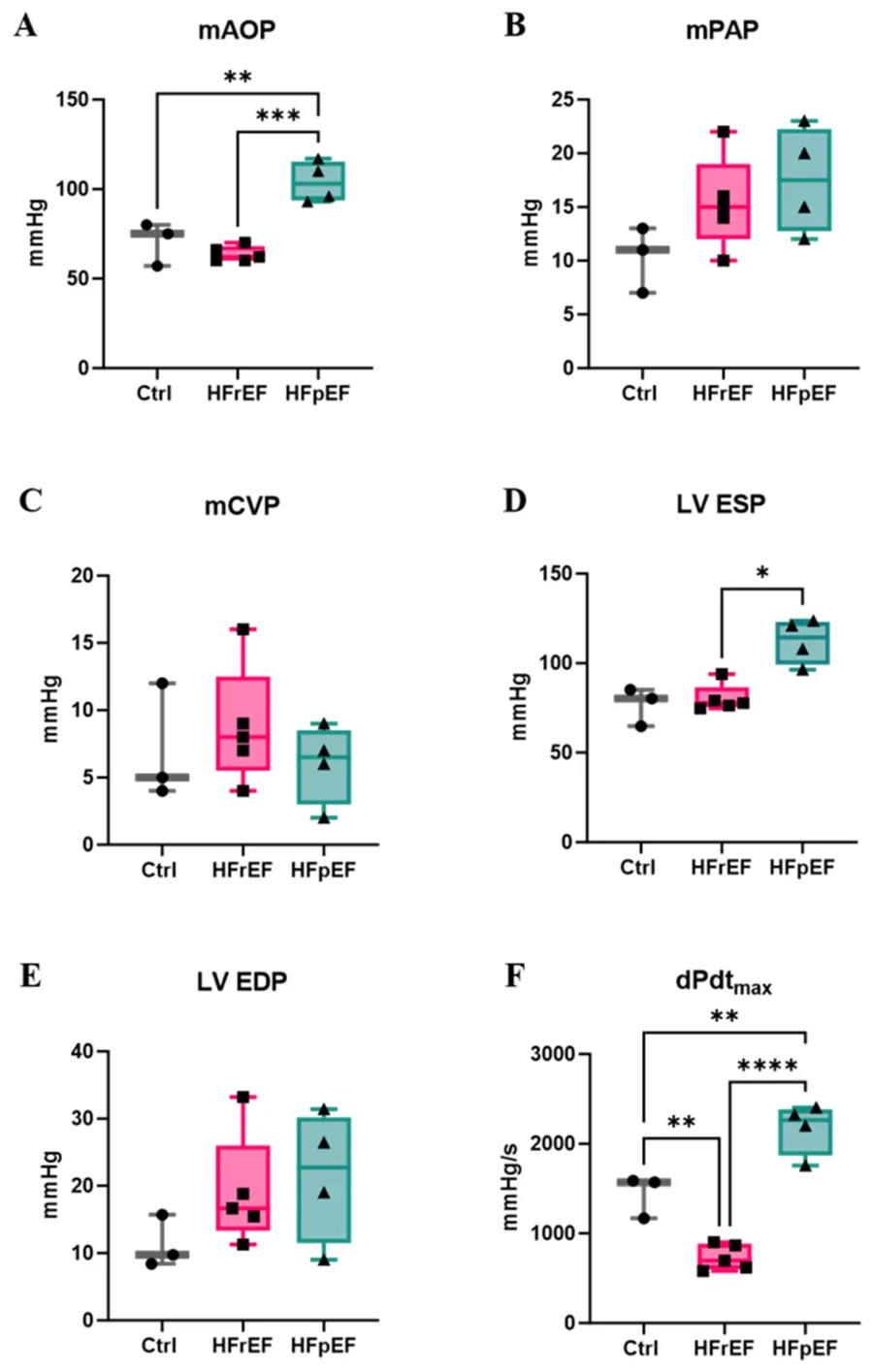
Hemodynamic profile of HFrEF and HFpEF porcine models. Boxplot of the hemodynamic parameters: (**A**) mean aortic pressure (mAOP); (**B**) mean pulmonary arterial pressure (mPAP); (**C**) mean central venous pressure (mCVP); (**D**) end-systolic pressure (LV ESP); (**E**) end-diastolic pressure (LV EDP); (**F**) maximum rate of pressure rise (dP/dt_max_). Comparisons are shown between healthy controls (Ctrl, grey), HFrEF (pink), and HFpEF (teal) minipig models. The HFpEF is characterized by a high-pressure phenotype with significantly elevated mAOP and LV ESP compared to the HFrEF model. Right-sided pressures (mPAP and mCVP) remained stable across all groups. Although not significant, both models show elevated LV EDP as a hallmark of heart failure. dP/dt_max_ suggests impaired systolic function in HFrEF. Data are expressed as median and interquartile range (25th–75th percentiles) with individual data points plotted. One-way ANOVA followed by the Šídák post hoc test for multiple comparisons with the exception of LV ESP in which the Kruskal–Wallis with Dunn’s multiple comparisons test was employed. * *p* < 0.05, ** *p* < 0.01, *** *p* < 0.001, **** *p* < 0.0001.

Baseline echocardiographic and invasive hemodynamic characteristics confirmed distinct structural and functional phenotypes across Control, HFrEF, and HFpEF groups (Figure 1 and Figure 2). Control animals served as physiological reference subjects for comparison with disease phenotypes. Furthermore, an extensive characterization of the HFrEF model has already been discussed in our previous work [18], while here we emphasize the differences compared to the HFpEF model.

No differences in heart rate (HR) were observed across the three groups (Figure 1A). While HFrEF showed a slightly lower average (71.00 ± 8.97 bpm), the variance suggests the heart rate remains relatively stable and comparable with Control and HFpEF (83.33 ± 4.50 bpm and 74.25 ± 11.44 bpm, respectively). As expected by the models, the ejection fraction (EF) was drastically reduced in HFrEF compared to both groups (Figure 1B), while HFpEF was comparable to Ctrl (Ctrl: 59% ± 2.5%; HFrEF: 23% ± 4.9%; HFpEF: 58.25% ± 3.30%). The LV internal diameter at diastole (LVIDd), a measure of chamber size, showed no significant difference between the groups (Figure 1C). This is in accordance with both HFrEF and HFpEF phenotypes, as dilation usually occurs at a later stage of “systolic” HF (39.90 mm ± 3.59 mm), while chamber size is normal or slightly reduced in “diastolic” HF (39.00 mm ± 5.59 mm). LV posterior wall thickness at diastole (LVPWd, Figure 1D) was increased in HFpEF compared to both Ctrl and HFrEF (Ctrl: 5.83 ± 1.04; HFrEF: 4.44 ± 0.62; HFpEF: 10.75 ± 0.95). The same trend was observed for the relative wall thickness (RWT, Figure 1E), with HFpEF showing higher values compared to both Ctrl and HFrEF (Ctrl: 0.37 ± 0.12; HFrEF: 0.22 ± 0.02; HFpEF: 0.55 ± 0.06). These findings indicate concentric hypertrophy in HFpEF, as compared to the eccentric remodeling (i.e., thin walls) that later occurs in HFrEF.

The invasive hemodynamic data acquired under anesthesia provided a deeper look into the physiological state of the porcine models, focusing on pressures and a surrogate of contractility (Figure 2).

The HFpEF group showed a significantly higher mean aortic pressure (mAOP) compared to both Ctrl and HFrEF (104.0 mmHg ± 11.40 mmHg vs. 70.67 mmHg ± 12.10 mmHg and vs. 63.60 mmHg ± 4.33 mmHg, respectively). This parameter characterizes HFpEF as a hypertensive phenotype, while HFrEF remains similar to healthy Control levels (Figure 2A). The mean pulmonary arterial pressure (mPAP) showed a slight upward trend in both HF models compared to the Control (Figure 2B), but the differences were not statistically significant, suggesting that these models have not yet developed advanced pulmonary hypertension. The mean central venous pressure (mCVP) was variable but similar across the groups (Figure 2C). The end-systolic pressure (LV ESP) was significantly elevated in the HFpEF group compared to the HFrEF group (112.3 mmHg ± 12.58 mmHg vs. 76.76 mmHg ± 10.62 mmHg). This confirms that the HFpEF model is driven by a high-pressure, hypertensive condition during contraction (Figure 2D). Although not significant, both HFrEF and HFpEF showed a trend toward an increased end-diastolic pressure (LV EDP) compared to controls (Figure 2E), supporting that both models are experiencing elevated back-pressure in the heart (i.e., a hallmark of HF). Noteworthy, on average, both models exceed the 15 mmHg threshold for a diagnosis of HF [2]. Finally, the maximum rate of pressure rise (dP/dt_max_), a surrogate of the systolic function, showed a marked decrease in HFrEF (732.9 mmHg/s ± 143.8 mmHg/s) and a significant increase in HFpEF (2172 mmHg/s ± 287.3 mmHg/s) with respect to Ctrl (1443 mmHg/s ± 237.7 mmHg/s). This distinct trend supports the fact that HFrEF has a weakened pump function, while reflecting the preserved and often hyperdynamic contractile state of the HFpEF heart (Figure 2F).

### 3.2. Prediction of Invasive LV Pressure Indices

**Figure 3:**
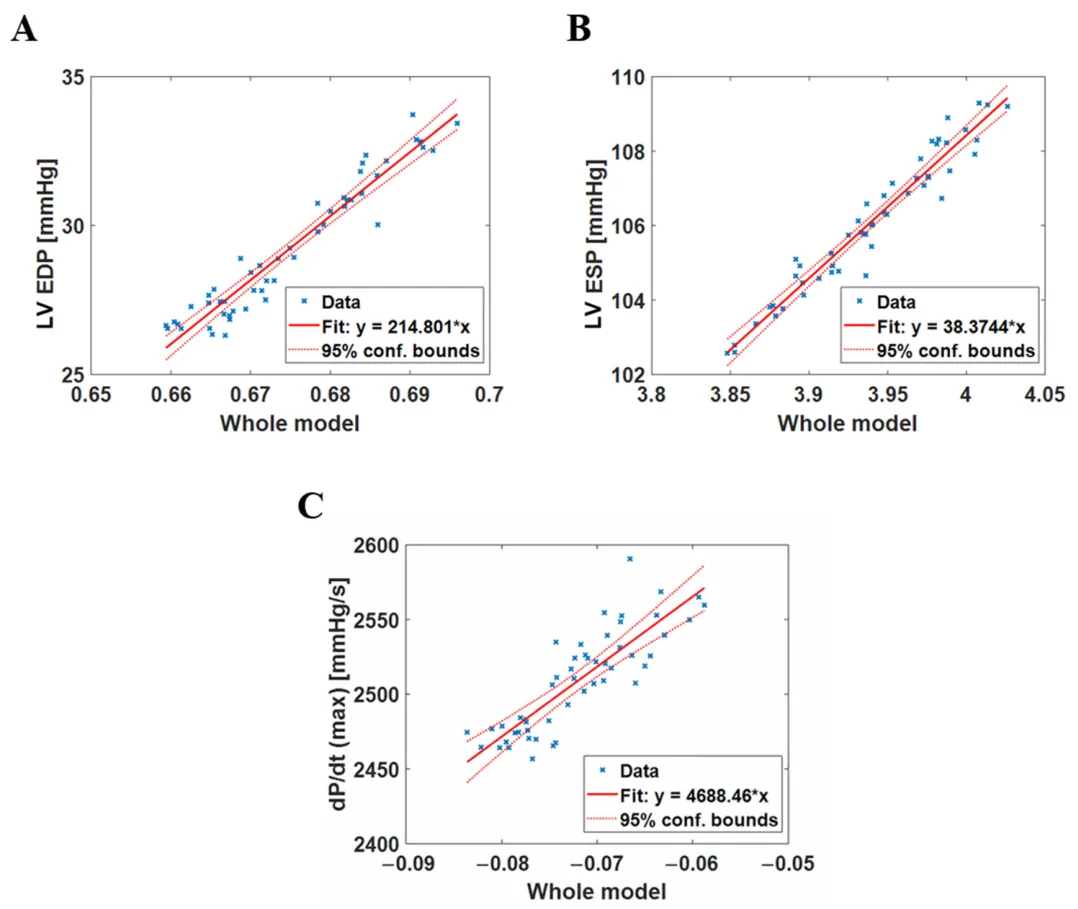
Representative regression analysis in HFpEF to predict LV functional indices. The linear regression is calculated at a spontaneous heart rate (HR) of ca. 80 bpm for (**A**) Left Ventricle end-diastolic pressure (LV EDP), with R^2^ = 0.925 and RMSE = 0.770 mmHg; (**B**) LV end-systolic pressure (LV ESP), with R^2^ = 0.939 and RMSE = 0.566 mmHg; (**C**) maximum rate of pressure rise (dP/dt_max_), with R^2^ = 0.724 and RMSE = 22.224 mmHg/s. The whole model (*x* axis) is the linear combination of the melody spectrum (MEL) features of the cardiac sounds plus the multiplicative inverse of the pulse transit time (invPTT). The blue crosses represent the analyzed heartbeats, the red line is the linear regression fit to predict the LV functional indices, and the red dotted curves are the 95% confidence bounds for the linear regression.

Similarly to our proof-of-concept study in the HFrEF model [15], Figure 3 shows the linear regression analysis in a representative HFpEF animal at a spontaneous HR of ca. 80 bpm, where the “whole model” (i.e., the predicting and non-invasive features condensed in their linear combination) predicts three invasively measured LV functional indices. Specifically, the use of the MEL features (i.e., set A plus set B, separately for diastole and systole) and invPTT showed a good prediction ability for LV EDP (Figure 3A, R^2^ = 0.925 and RMSE = 0.770 mmHg), ESP (Figure 3B, R^2^ = 0.939 and RMSE = 0.566 mmHg), and dP/dt_max_ (Figure 3C, R^2^ = 0.724 and RMSE = 22.224 mmHg/s), with relative prediction errors below 3% in this experimental setting. These findings indicate a strong association between time-frequency features and invasively measured LV pressure indices in HFpEF minipigs.

Moreover, the model’s performance was preserved even during the increasing HR protocol (i.e., 100, 120, 140, and 160 bpm) for all HFpEF animals (*n* = 4).

Table 1 summarizes these results for LV EDP, LV ESP, and dP/dt_max_ at each pacing step, indicating that the relationship between acoustic features and LV functional indices remained stable under heart-rate stress.

### 3.3. Differences in Acoustic Cardiography and invPTT Features

**Figure 4:**
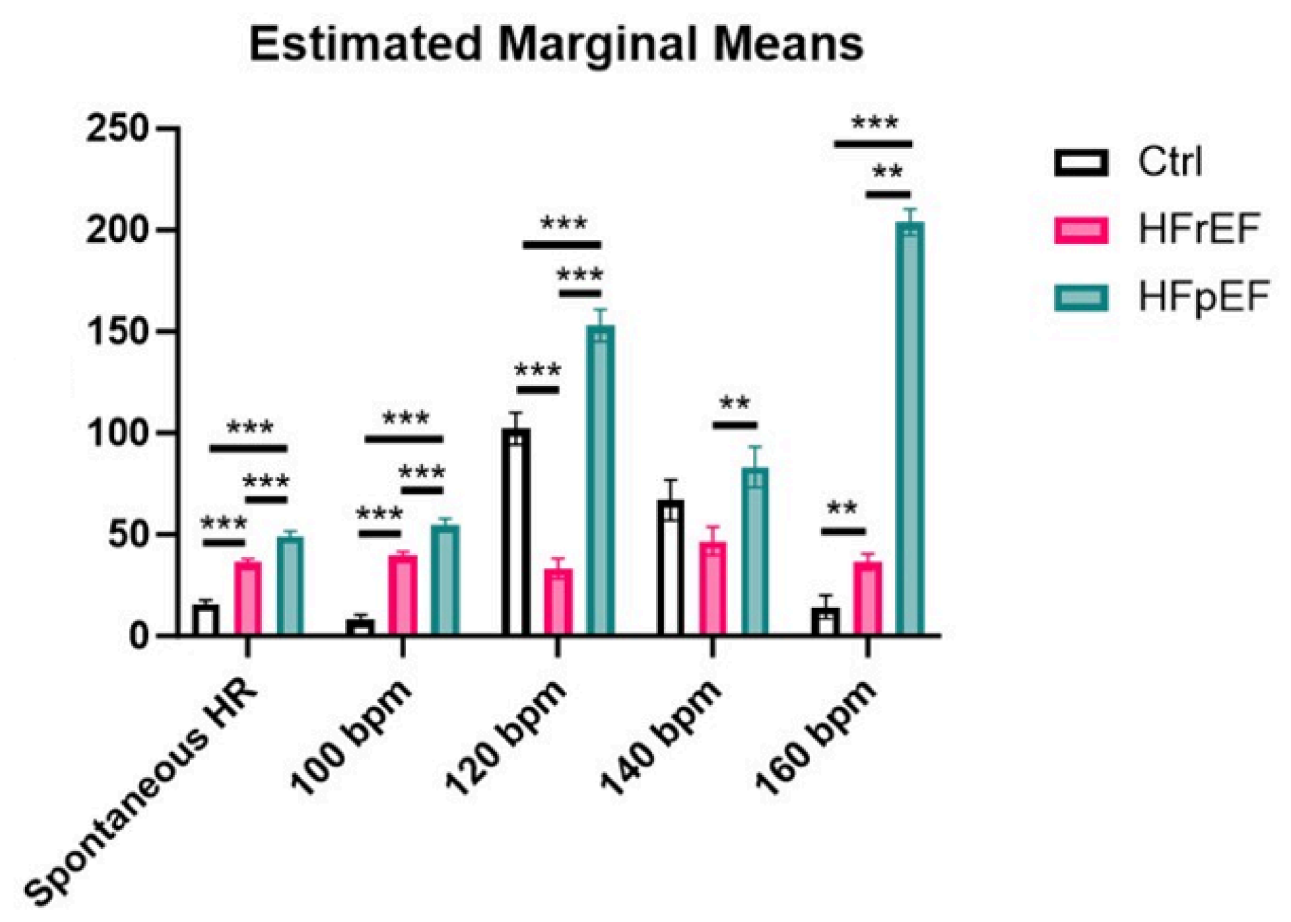
Predicting features comparison across the three health statuses. The estimated marginal means with their standard errors were calculated for each health status (i.e., Control, HFpEF, and HFrEF) at each heart rate (spontaneous HR, 100 bpm, 120 bpm, 140 bpm, and 160 bpm). Data are expressed as mean ± SD. A multivariate repeated measures ANOVA with the estimated marginal means followed by Tukey–Kramer post hoc test was performed. This test isolates the effect of the primary factor of interest (i.e., the health status or the HR) from the confounding influence of other variables. An α of 0.05 was elected as significant. ** *p* < 0.01, *** *p* < 0.001. In the figure, only the inter-group results are highlighted. The details of the statistical analysis are reported in Supplemental Material Tables S1 and S2.

To compare phenotypic differences across heart rates, the estimated marginal means of the predicting features were evaluated for each study group, i.e., Control, HFpEF, and HFrEF, using the AC predictors of both diastole and systole plus the invPTT (i.e., a total of 35 predicting features). Notably, HFpEF animals demonstrated greater variability in estimated marginal means compared to both Control and HFrEF (Figure 4). This variability became more pronounced by increasing HR following the atrial pacing protocol. In contrast, the HFrEF animals showed a comparatively constrained response, while Control animals demonstrated high variability. In particular, the comparisons between the three groups were significant across all HRs, except for 140 bpm where only HFrEF vs. HFpEF was significant. Due to the multivariate comparisons, Figure 4 highlights only the inter-group results. Supplemental Material Tables S1 and S2 report the detailed results of the statistical analysis across all HRs for each group.

### 3.4. Artificial Intelligence Distinguishes the HF Phenotypes

The two abovementioned supervised classification strategies (i.e., 80/20 and leave-one-out) were evaluated to assess whether the acoustic cardiography-derived features plus the invPTT support the phenotypic differentiation in experimental HF. In our first approach, using all the minipigs from the three groups, the AI models showed validation accuracy and test accuracy both greater than 95% (Table 2 with the top 3 results for each HR).

When evaluated using the leave-one-animal-out for training and validation, the classification performance remained robust, with a validation accuracy greater than 90% and a test accuracy greater than 79% (Table 3, with the top 3 results for each HR), supporting generalizability at the subject level despite inter-animal variability. Classification performance was maintained across increasing heart rates, indicating that the phenotypic discrimination persisted under stress conditions. Across analyses, findings were consistent when accounting for repeated measurements within animals, with the animal treated as the unit of biological replication.

## 4. Discussion

In this study, we extend our previous proof-of-concept work in HFrEF [15] to a clinically relevant porcine model of cardiometabolic HFpEF and demonstrate that multimodal acoustic cardiography combined with artificial intelligence can non-invasively predict left-ventricular-pressure-derived indices and discriminate HF phenotypes under controlled HR stress. While HFrEF represents a relatively homogeneous syndrome defined by impaired systolic pump function, HFpEF remains diagnostically challenging due to its heterogeneity and stress-dependent hemodynamic abnormalities. By integrating invasive hemodynamics with synchronized acoustic, ECG, and pulse oximetry signals, we show that time–frequency features extracted from cardiac sounds contain sufficient information not only to estimate LV functional indices but also to capture phenotype-specific mechanical signatures across increasing heart rates HRs. Together, these findings support the concept that advanced AC analysis may serve as a scalable tool for functional phenotyping rather than a replacement for established diagnostic pathways and protocols.

### 4.1. Experimental HF Models

The two experimental models used in this study were selected to represent mechanistically distinct HF phenotypes. The tachypacing-induced HFrEF model [18] reproduces a homogeneous systolic dysfunction characterized by reduced contractility and eccentric remodeling, whereas the two-hit cardiometabolic HFpEF model integrates obesity, hypertension, and concentric remodeling, recapitulating key features of the human syndrome [29,30]. Our echocardiographic and invasive hemodynamic data confirmed these divergent phenotypes, with depressed dP/dt_max_ and reduced EF in HFrEF, whilst preserved EF with elevated systolic pressures and concentric hypertrophy in HFpEF. This clear phenotypic separation provided a controlled framework to test whether acoustic cardiography–derived features reflect underlying biomechanical differences rather than merely global cardiac dysfunction.

### 4.2. Acoustic Cardiography Features Depend on HF Phenotypes

Our method combined both time (i.e., invPTT) and frequency features (i.e., set A and set B from the melody spectrum), an approach known to improve the quality of the results when working with AC [27,28,31]. The linear regression models achieved high performance in predicting LV EDP, LV ESP, and dP/dt_max_ in the HFpEF animals, with relative errors below 3%. This finding was in accordance with our previous study in HFrEF, suggesting that the spectral properties of the heart sounds encapsulate valuable insights into the underlying hemodynamic conditions.

Moreover, we wanted to investigate whether there was any difference in time-frequency features between HFpEF, HFrEF, and Control animals. Interestingly, the distribution of the estimated marginal means across HRs revealed phenotype-specific trends. In fact, HFpEF animals exhibited greater variability in time-frequency features compared to both healthy controls and HFrEF animals. Consequently, we hypothesize that this finding reflects the heterogeneous diastolic impairment and the compensatory mechanisms characteristic of cardiometabolic HF [32,33]. On the other hand, HFrEF animals displayed less dispersed estimated marginal means due to a more homogenous phenotype of contractile dysfunction and cardiac remodeling. We believe these trends reveal that the combined use of time and frequency predicting features yields the ability to unmask subtle variations in myocardial stiffness, chamber compliance, and mechanical variability intrinsic of the specific HF phenotype, which may not be directly visible through standard AC analysis.

### 4.3. Performance of AI Classifiers

Investigating the possibility of the AC-derived features to be HF-specific, we built different machine learning classifiers to recognize whether a specific set of time and frequency features could distinguish between Control, HFrEF, and HFpEF. The first classifiers were built with 10-fold cross-validation with a standard 80/20 train/test ratio, achieving high validation (>95%) and test (>95%) accuracies, although these results should be cautiously interpreted given the limited sample size. Our second approach was built following 10-fold cross-validation with the leave-one-out strategy (i.e., 1 animal per health status) during training and validation, not only to avoid possible overfitting but also to simulate a real-world diagnostic scenario. These second classifiers showed a validation accuracy greater than 90%, whereas the test accuracy with the remaining 3 animals was greater than 79%. These findings suggest potential reproducibility at the subject level, although confirmation in larger and more heterogeneous cohorts is required. Moreover, the consistency of the classification across increasing HR suggests that the models capture disease-specific patterns that persist under physiological stress, supporting further investigation of this approach in clinically relevant stress-testing paradigms, which is especially relevant for HFpEF [6].

### 4.4. Limitations

Several limitations should be acknowledged. First, the study included a limited and unequal number of animals derived from two experimental protocols, which may restrict generalizability. Second, all animals were female, representing an experimental compromise (i.e., easier to handle than males) but potentially limiting extrapolation across sexes. Third, anesthesia and controlled pacing do not replicate the systemic complexity of exercise stress in humans. Fourth, transmitral E/A ratio, tissue-Doppler E/e′, and left atrial volume provide additional information regarding diastolic function, which is especially useful for HFpEF. However, these measurements were not acquired with sufficient consistency and image quality to permit reliable analysis in the present cohort. In particular, reproducible Doppler alignment and left atrial volumetric assessment are technically challenging in swine because of the orientation of the porcine heart and the limited availability of standardized acoustic windows. Fifth, echocardiographic measurements were obtained by a single operator, and interobserver variability could therefore not be assessed. However, echocardiography was used only for supportive phenotypic characterization, whereas the primary experimental classification and analyses were based on direct invasive left ventricular pressure measurements and acoustic cardiography.

Finally, although porcine cardiac physiology closely resembles human physiology, translation to clinical application will require validation in larger and heterogeneous patient populations. Therefore, our findings should be interpreted as mechanistic and hypothesis-generating rather than diagnostic validation.

## 5. Conclusions

In summary, this study demonstrates that multimodal acoustic cardiography combined with artificial intelligence can non-invasively predict LV-pressure-derived indices and discriminate heart failure phenotypes in mechanistically distinct porcine models and under heart-rate stress. By capturing phenotype-specific mechanical variability, this approach highlights the potential of advanced heart sound analytics to support functional phenotyping, particularly in conditions such as HFpEF where stress-dependent abnormalities are central to diagnosis. Further clinical validation will determine whether this strategy can evolve into a scalable adjunct to contemporary heart failure assessment.

## Figures and Tables

**Table 1:** Results of the linear regression analysis for the HFpEF animals. The predictors are the MEL features of the cardiac sounds (set A plus set B) together with the invPTT for all HFpEF animals (*n* = 4). The unit of measurement for RMSE is the same as the related predicted parameter. Prediction performance was elevated across increasing HR.

HFpEF			
Pacing	To Be Predicted	R^2^	RMSE
Spontaneous HR	LV EDP	0.925	0.770
Spontaneous HR	LV ESP	0.939	0.566
Spontaneous HR	dP/dt_max_	0.724	22.224
100 bpm	LV EDP	0.869	0.825
100 bpm	LV ESP	0.855	0.805
100 bpm	dP/dt_max_	0.611	17.994
120 bpm	LV EDP	0.947	0.678
120 bpm	LV ESP	0.930	0.727
120 bpm	dP/dt_max_	0.641	27.173
140 bpm	LV EDP	0.890	0.586
140 bpm	LV ESP	0.813	0.951
140 bpm	dP/dt_max_	0.776	32.878
160 bpm	LV EDP	0.934	0.448
160 bpm	LV ESP	0.902	1.154
160 bpm	dP/dt_max_	0.971	22.440

MEL: melody; invPTT: inverse of pulse transit time; HFpEF: heart failure with preserved ejection fraction; RMSE: root mean square error; HR: heart rate; bpm: beats per minute; LV EDP: end-diastolic pressure; LV ESP: end-systolic pressure; dP/dt_max_: maximum rate of pressure rise.

**Table 2:** First AI model approach and results. All the animals were used to train, validate, and test the AI models. In detail, to classify an observation into HFpEF, HFrEF, or Control, we employed all the features of the diastolic and systolic cardiac sounds plus the invPTT (i.e., 35 features in total); the 80% of the dataset was used to perform the AI models’ training via 10-fold cross-validation, whilst the remaining 20% was used to blindly test the trained and validated models. For reproducibility, all the hyperparameters of the AI models are also reported.

Pacing	AI Model	Validation Accuracy (%)	Test Accuracy (%)	Hyperparameters
Spontaneous HR	Neural Network	99.18	99.59	Number of fully connected layers: 1; First layer size: 25; Activation: ReLU; Iteration limit: 1000; Regularization strength (Lambda): 0; Standardize data: Yes
Spontaneous HR	SVM	99.18	99.18	Kernel function: Quadratic; Kernel scale: Automatic; Box constraint level: 1; Multiclass coding: One-vs-One; Standardize data: Yes
Spontaneous HR	SVM	99.08	99.18	Kernel function: Cubic; Kernel scale: Automatic; Box constraint level: 1; Multiclass coding: One-vs-One; Standardize data: Yes
100 bpm	SVM	98.47	98.11	Kernel function: Quadratic; Kernel scale: Automatic; Box constraint level: 1; Multiclass coding: One-vs-One; Standardize data: Yes
100 bpm	SVM	98.23	98.11	Kernel function: Cubic; Kernel scale: Automatic; Box constraint level: 1; Multiclass coding: One-vs-One; Standardize data: Yes
100 bpm	Ensemble	98.82	97.64	Ensemble method: RUSBoost; Learner type: Decision tree; Maximum number of splits: 20; Number of learners: 30; Learning rate: 0.1; Number of predictors to sample: Select All
120 bpm	SVM	98.69	100	Kernel function: Quadratic; Kernel scale: Automatic; Box constraint level: 1; Multiclass coding: One-vs-One; Standardize data: Yes
120 bpm	Neural Network	98.54	100	Number of fully connected layers: 2; First layer size: 10; Second layer size: 10; Activation: ReLU; Iteration limit: 1000; Regularization strength (Lambda): 0; Standardize data: Yes
120 bpm	Discriminant	97.96	99.41	Covariance structure: Full
140 bpm	SVM	97.17	97.64	Kernel function: Cubic; Kernel scale: Automatic; Box constraint level: 1; Multiclass coding: One-vs-One; Standardize data: Yes
140 bpm	Kernel	96.35	97.64	Learner: SVM; Number of expansion dimensions: Auto; Regularization strength (Lambda): Auto; Kernel scale: Auto; Multiclass coding: One-vs-One; Standardize data: Yes; Iteration limit: 1000
140 bpm	Ensemble	96.82	97.16	Ensemble method: Bag; Learner type: Decision tree; Maximum number of splits: 850; Number of learners: 30; Number of predictors to sample: Select All
160 bpm	KNN	97.12	97.12	Number of neighbors: 1; Distance metric: Euclidean; Distance weight: Equal; Standardize data: Yes
160 bpm	Neural Network	96.58	97.12	Number of fully connected layers: 1; First layer size: 25; Activation: ReLU; Iteration limit: 1000; Regularization strength (Lambda): 0; Standardize data: Yes
160 bpm	Neural Network	96.94	97.12	Number of fully connected layers: 1; First layer size: 100; Activation: ReLU; Iteration limit: 1000; Regularization strength (Lambda): 0; Standardize data: Yes

HR: heart rate; bpm: beat per minute; SVM: support vector machine; KNN: *k*-nearest neighbor.

**Table 3:** Second AI model approach and results. All the animals, with the exception of three subjects (i.e., 1 HFpEF, 1 HFrEF, 1 Control), were used to train and validate the AI models. In detail, to classify an observation into HFpEF, HFrEF, or Control, we employed all the features of the diastolic and systolic cardiac sounds plus the invPTT (i.e., 35 features in total); the training dataset was used to perform the AI models’ training via 10-fold cross-validation, whilst the remaining three animals were utilized to blindly test the trained and validated models. For reproducibility, all the hyperparameters of the AI models are also reported.

Pacing	AI Model	Validation Accuracy (%)	Test Accuracy (%)	Hyperparameters
Spontaneous HR	Ensemble	99.42	94.64	Ensemble method: RUSBoost; Learner type: Decision tree; Maximum number of splits: 20; Number of learners: 30; Learning rate: 0.1; Number of predictors to sample: Select All
Spontaneous HR	SVM	98.43	92.54	Kernel function: Cubic; Kernel scale: Automatic; Box constraint level: 1; Multiclass coding: One-vs-One; Standardize data: Yes
Spontaneous HR	Ensemble	99.00	89.48	Ensemble method: Bag; Learner type: Decision tree; Maximum number of splits: 751; Number of learners: 30; Number of predictors to sample: Select All
100 bpm	Tree	99.60	90.96	Maximum number of splits: 4; Split criterion: Gini’s diversity index; Surrogate decision splits: Off
100 bpm	Ensemble	99.33	90.64	Ensemble method: Bag; Learner type: Decision tree; Maximum number of splits: 751; Number of learners: 30; Number of predictors to sample: Select All
100 bpm	Ensemble	99.46	88.38	Ensemble method: RUSBoost; Learner type: Decision tree; Maximum number of splits: 20; Number of learners: 30; Learning rate: 0.1; Number of predictors to sample: Select All
120 bpm	Ensemble	99.30	95.62	Ensemble method: Subspace; Learner type: Discriminant; Number of learners: 30; Subspace dimension: 18
120 bpm	SVM	98.06	92.70	Kernel function: Gaussian; Kernel scale: 24; Box constraint level: 1; Multiclass coding: One-vs-One; Standardize data: Yes
120 bpm	Discriminant	98.75	91.97	Covariance structure: Full
140 bpm	SVM	90.02	87.28	Kernel function: Gaussian; Kernel scale: 24; Box constraint level: 1; Multiclass coding: One-vs-One; Standardize data: Yes
140 bpm	Ensemble	91.96	84.53	Ensemble method: Subspace; Learner type: Discriminant; Number of learners: 30; Subspace dimension: 18
140 bpm	Neural Network	96.50	82.13	Number of fully connected layers: 1; First layer size: 100; Activation: ReLU; Iteration limit: 1000; Regularization strength (Lambda): 0; Standardize data: Yes
160 bpm	Ensemble	96.36	85.77	Ensemble method: Subspace; Learner type: Discriminant; Number of learners: 30; Subspace dimension: 18
160 bpm	Discriminant	94.65	82.52	Covariance structure: Full
160 bpm	Kernel	94.44	79.26	Learner: SVM; Number of expansion dimensions: Auto; Regularization strength (Lambda): Auto; Kernel scale: Auto; Multiclass coding: One-vs-One; Standardize data: Yes; Iteration limit: 1000

HR: heart rate; bpm: beat per minute; SVM: support vector machine.

## Data Availability

The raw signal data were acquired using ADInstruments LabChart software and exist in large, proprietary formats (.*adicht*) that require specific software and version compatibility for proper visualization and analysis. To ensure that recipients receive the correct file versions alongside the necessary technical metadata and channel configurations for proper interpretation, the raw datasets are maintained on institutional servers and will be shared promptly upon direct request to the corresponding authors.

## Supplementary Materials

The following supporting information can be downloaded at: https://www.mdpi.com/article/10.3390/biomedicines14081669/s1, Table S1: Estimated marginal means inter-group comparisons between Control, HFrEF, and HFpEF; Table S2: Estimated marginal means intra-group comparisons between Control, HFrEF, and HFpEF; Figure S1: Multicollinearity between features for HFpEF animals.

## Abbreviations

The following abbreviations are used in this manuscript:

HF	Heart Failure
HFrEF	Heart Failure with Reduced Ejection Fraction
HFpEF	Heart Failure with Preserved Ejection Fraction
AI	Artificial Intelligence
ML	Machine Learning
LV EDP	Left Ventricle End-Diastolic Pressure
ESP	Left Ventricle End-Systolic Pressure
dP/dt_max_	maximum rate of pressure rise
MIC	Microphone signal
POX	Pulse Oximetry signal
MED	Microphone Envelope Difference signal
MEL	Melody spectrum
invPTT	Multiplicative inverse of Pulse Transit Time
