# Supplementary material for: Acoustic Cardiography Captures Stress-Dependent Ventricular Dysfunction and Distinguishes Heart Failure Phenotypes in a Porcine Model
Source: Biomedicines. 2026 Jul 24;14(8):1669. doi: 10.3390/biomedicines14081669 (PMC13509373; doi:10.3390/biomedicines14081669)
Supplement: Supplementary file 1 [file biomedicines-14-01669-s001.zip › Figure S1.pdf]

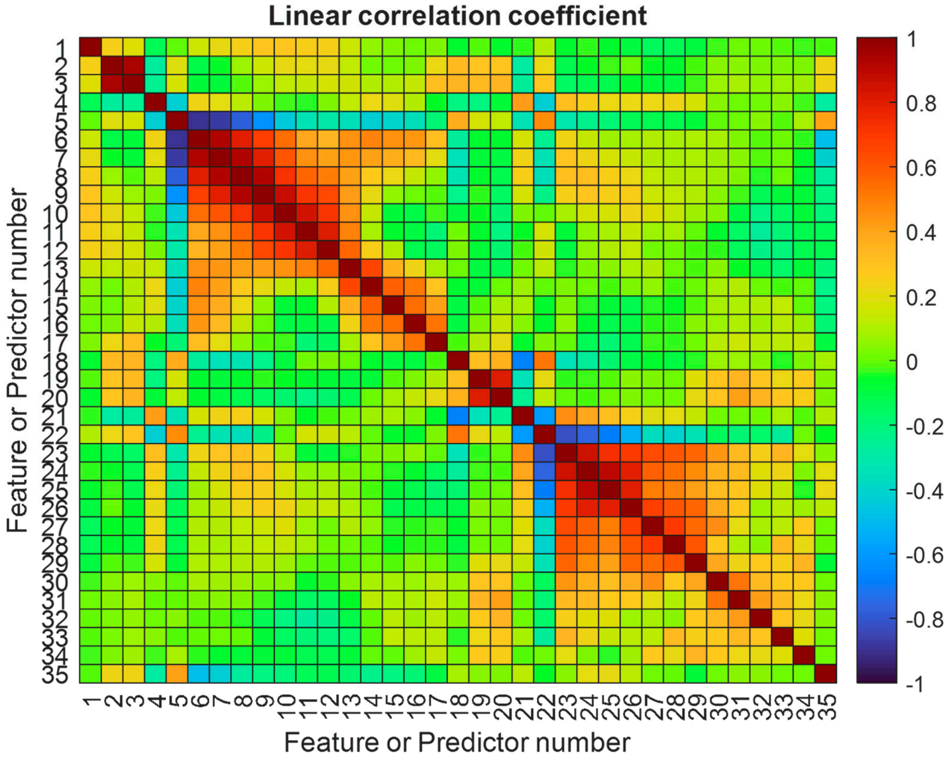

Supplemental Figure S1. Multicollinearity between features for HFpEF animals. The first 34 features are the acoustic cardiography predictors of both systole and diastole, whereas the 35th is the invPTT. Overall, no strong pairwise correlations were observed, suggesting that immediate pairwise redundancy was not a major concern for the model. However, a limited number of acoustic features show collinearity but they are usually used together to describe an acoustic phenomenon so we opted to not exclude them *a priori*. More importantly, the invPTT (i.e. arising from ECG and POX signals) was found not correlated with the acoustic features. Both Ctrl and HFrEF showed similar behavior (data not shown).
