# Supplementary material for: Acoustic Cardiography Captures Stress-Dependent Ventricular Dysfunction and Distinguishes Heart Failure Phenotypes in a Porcine Model
Source: Biomedicines. 2026 Jul 24;14(8):1669. doi: 10.3390/biomedicines14081669 (PMC13509373; doi:10.3390/biomedicines14081669)
Supplement: Supplementary file 1 [file biomedicines-14-01669-s001.zip › Table S1.pdf]

Supplemental Table S1. Estimated marginal means inter-group comparisons between Control, HFrEF, and HFpEF. Estimated marginal means were calculated using acoustic cardiography predictors of both diastole and systole plus the invPTT (i.e., a total of 35 predicting features). Displayed are the results of the multivariate repeated measures ANOVA followed by the Tukey's Honest Significant Difference *post-hoc* test, isolating the effect of the primary factor of interest (i.e., the clinical health status) from the confounding influence of other variables.

**Atrial pacing steps**	**Class 1**	**Class 2**	**p-value**
Spontaneous HR	HFpEF	HFrEF	<0.001
	HFpEF	Ctrl	<0.001
	HFrEF	Ctrl	<0.001
100 bpm	HFpEF	HFrEF	<0.001
	HFpEF	Ctrl	<0.001
	HFrEF	Ctrl	<0.001
120 bpm	HFpEF	HFrEF	<0.001
	HFpEF	Ctrl	<0.001
	HFrEF	Ctrl	<0.001
140 bpm	HFpEF	HFrEF	0.008
	HFpEF	Ctrl	0.496
	HFrEF	Ctrl	0.244
160 bpm	HFpEF	HFrEF	<0.001
	HFpEF	Ctrl	<0.001
	HFrEF	Ctrl	0.004

[1] Ctrl: control; [2] HFrEF: heart failure with reduced ejection fraction; [3] HFpEF: heart failure with preserved ejection fraction; [4] invPTT: multiplicative inverse of pulse transit time; [5] HR: heart rate; [6] bpm: beats per minute.
