# Supplementary material for: Acoustic Cardiography Captures Stress-Dependent Ventricular Dysfunction and Distinguishes Heart Failure Phenotypes in a Porcine Model
Source: Biomedicines. 2026 Jul 24;14(8):1669. doi: 10.3390/biomedicines14081669 (PMC13509373; doi:10.3390/biomedicines14081669)
Supplement: Supplementary file 1 [file biomedicines-14-01669-s001.zip › Table S2.pdf]

**Supplemental Table S2.** Estimated marginal means intra-group comparisons between Control, HFrEF, and HFpEF. Estimated marginal means were calculated using acoustic cardiography predictors of both diastole and systole plus the invPTT (i.e., a total of 35 predicting features). Displayed are the results of the multivariate repeated measures ANOVA followed by the Tukey's Honest Significant Difference *post-hoc* test, isolating the effect of the primary factor of interest (i.e., the HR) from the confounding influence of other variables.

| Phenotype | Class 1 | Class 2 | p-value |
|---|---|---|---|
| Ctrl | Spontaneous HR | 100 bpm | 0.899 |
| | Spontaneous HR | 120 bpm | <0.001 |
| | Spontaneous HR | 140 bpm | <0.001 |
| | Spontaneous HR | 160 bpm | 0.999 |
| | 100 bpm | 120 bpm | <0.001 |
| | 100 bpm | 140 bpm | <0.001 |
| | 100 bpm | 160 bpm | 0.977 |
| | 120 bpm | 140 bpm | 0.009 |
| | 120 bpm | 160 bpm | <0.001 |
| | 140 bpm | 160 bpm | <0.001 |
| HFrEF | Spontaneous HR | 100 bpm | 0.935 |
| | Spontaneous HR | 120 bpm | 0.952 |
| | Spontaneous HR | 140 bpm | 0.096 |
| | Spontaneous HR | 160 bpm | 0.999 |
| | 100 bpm | 120 bpm | 0.596 |
| | 100 bpm | 140 bpm | 0.438 |
| | 100 bpm | 160 bpm | 0.963 |
| | 120 bpm | 140 bpm | 0.020 |
| | 120 bpm | 160 bpm | 0.960 |
| | 140 bpm | 160 bpm | 0.186 |
| HFpEF | Spontaneous HR | 100 bpm | 0.991 |
| | Spontaneous HR | 120 bpm | <0.001 |
| | Spontaneous HR | 140 bpm | 0.034 |
| | Spontaneous HR | 160 bpm | <0.001 |
| | 100 bpm | 120 bpm | <0.001 |
| | 100 bpm | 140 bpm | 0.112 |
| | 100 bpm | 160 bpm | <0.001 |
| | 120 bpm | 140 bpm | <0.001 |
| | 120 bpm | 160 bpm | 0.005 |
| | 140 bpm | 160 bpm | <0.001 |

[1] Ctrl: control; [2] HFrEF: heart failure with reduced ejection fraction; [3] HFpEF: heart failure with preserved ejection fraction; [4] invPTT: multiplicative inverse of pulse transit time; [5] HR: heart rate; [6] bpm: beats per minute.
